# Exposure assessment and risks associated with wearing silver nanoparticle-coated textiles

**DOI:** 10.12688/openreseurope.17254.2

**Published:** 2024-10-02

**Authors:** Antti Joonas Koivisto, David Burrueco-Subirà, Ana Candalija, Socorro Vázquez-Campos, Alessia Nicosia, Fabrizio Ravegnani, Irini Furxhi, Andrea Brigliadori, Ilaria Zanoni, Magda Blosi, Anna Costa, Franco Belosi, Jesús Lopez de Ipiña

**Affiliations:** 1 Air Pollution Management APM, Tampere, FI-33610, Finland; 2 University of Helsinki, Institute for Atmospheric and Earth System Research, Helsinki, FI-00014, Finland; 3 ARCHE Consulting, Wondelgem, B-9032, Belgium; 4 Leitat Technological Center, Barcelona, 08040, Spain; 5 National Research Council of Italy, Institute of Atmospheric Sciences and Climate, Bologna, 40129, Italy; 6 National Research Council of Italy, Institute of Science, Technology and Sustainability for Ceramics, Faenza, 48018, Italy; 7 TECNALIA Research and Innovation - Basque Research and Technology Alliance, Miñano, 01510, Spain

**Keywords:** Nanoparticles, dermal exposure, mass balance, release, dermal intake, risk characterization ratio, conditions of use, REACH

## Abstract

**Background:**

Silver (Ag) nanoparticles (NPs) are used increasingly in consumer and healthcare fabrics due to their antimicrobial properties. Abrasive leaching experiments have shown that AgNPs can be released during textile wear and cause a dermal exposure. Derived-no-effect-limit value for AgNPs ranges from 0.01 to 0.0375 mg/kg-body-weight, and thus, low exposures levels can cause relevant risk.

**Methods:**

In this study AgNP release from textiles by artificial sweat immersion and mechanical stress was investigated. A mass balance model was used to calculate dermal Ag exposure and potential intake via percutaneous absorption and inadvertent (peri-)oral intake during wear of face mask, suit with a full body exposure and gloves. Mass flow analysis was performed for up to 8-h wear time and by using Ag penetration rate constants reported for fresh-, cryopreserved- and glycerolized skin grafts.

**Results:**

Dermal intake risk characterization ratio (RCR) during 8-h wear time for glycerolized skin was up to 0.02 for face mask and 0.9 for full body wear in a worst-case condition. Wearing gloves for 1-h followed by single unintentional fingertip mouthing (contact area 11.5 cm ^2^) resulted in an RCR of 0.0002. RCR varied depending on the type of textile-product, exposure wear duration and skin type.

**Conclusions:**

This study provides a comprehensive assessment of AgNPs release from textiles and their potential impact on human dermal exposure and was essential for understanding the safety implications for different exposure scenarios and mitigating potential risks.

## Plain language summary

Silver (Ag) nanoparticles (NP) are widely used in textiles designed for general population and healthcare. However, there is ongoing research and concern about the potential health risks associated with the release of nanoparticles into the environment. Here, the risk was studied for face mask, gloves and a full body suit use scenarios and for different skin types and Ag NPs to identify realistic exposure scenarios that can lead to excessive risk. The major risk was associated with unintentional finger mouthing after wearing gloves containing AgNPs. However, the current release test settings are not designed for NP release assessment from textiles during realistic use scenarios. This study emphasizes following good hygiene practices when wearing Ag containing textiles to avoid intake via oral exposure.

## List of abbreviations

**Table:** 

ASINA	Anticipating Safety Issues at the Design Stage of NAno Product Development
Ag	Silver
NP	Nanoparticle
DNEL	Derived-No-Effect-Limit
RWC	Reasonable Worst-Case
RCR	Risk Characterization Ratio
AgCur	Silver nanoparticles, coupled with Curcumine
AgHEC6.4	Silver nanoparticles, embedded in cationic quaternized hydroxyethylcellulose matrix (DoE optimized molar ratio HEC/Ag:NaOH/Ag 6,4:1,4)
AgHEC	Silver nanoparticles, embedded in cationic quaternized hydroxyethylcellulose matrix (patented molar ratio HEC/Ag:NaOH/Ag 5,5:2,8)

## Introduction

Silver (Ag) nanoparticles (NPs) are used in antimicrobial textiles, such as face masks and gloves, personal clothing, and wound dressings for their ability to inhibit the growth of microorganisms ( Abazari *et al*., 2023; Novi *et al*., 2022; Salleh *et al.*, 2020). However, concerns have been raised regarding the potential intake via long term dermal exposure ( Shah *et al*., 2022; Ferdous & Nemmar, 2019; Yetisen *et al*., 2016). In response to these concerns, regulatory actions have been taken in Europe where the European Commission (EC) has restricted the use of Ag NPs as a biocidal substance in fiber, leather, rubber and polymerized materials preservatives ( EC, 2021). This regulatory measure reflects the need to mitigate potential risks associated with the widespread use of Ag NPs in consumer products.

Dermal exposure to NPs can occur via deposition from air to skin, intentional application of a product to skin or contact with articles containing NPs. NPs at the skin surface may be removed by volatilization, debridement, sweating, washing, and percutaneous absorption. Main pathways for dermal uptake are percutaneous absorption, direct absorption through damaged skin, or indirect uptake via inadvertent ingestion ( Anderson & Meade, 2014; Gorman Ng *et al*., 2016; Li *et al*., 2021). Indirect exposure pathways are for example hand-to-object contact followed by finger mouthing or object-to-mouth contact for example when wearing a face mask. Dermal exposure mechanisms are well known and various mechanistic dermal exposure models are developed (e.g., Ernstoff *et al*., 2016; Kvasnicka *et al*., 2020; Li *et al*., 2021).

The skin barrier is composed of the epidermis and dermis ( Nafisi & Maibach, 2018). The epidermis is made of keratinocytes and non-keratinocytes primary cells that are stratified in layers as *stratum corneum* (the outermost layer) and viable skin of *stratum lucidum*, *stratum granulosum*, *stratum spinosum* and *stratum basale* (on top of dermis). Intake via percutaneous absorption is usually considered the fraction that penetrate into *stratum corneum* ( Cleek & Bunge, 1993). From *stratum corneum*, the chemical can permeate to the viable skin and enter the systemic circulation that is considered as uptake. A simplified concept for dermal absorption across the *stratum corneum* is presented by van der Merwe *et al.* (2006). Chemical permeation is typically measured *in vitro* by using diffusion cell and skin samples with only *stratum corneum* and epidermis ( ECHA, 2020; OECD, 2004).

Currently, there are no sophisticated mathematical models for predicting absorption of NPs through different layers of the skin ( ECHA, 2020). This is because dermal absorption models are designed for neutral and low-molecular weight molecules whose dynamics are described with kinetic molecular theory which is not applicable for colloidal NP systems ( Praetorius *et al*., 2014). NP absorption becomes a complex phenomenon with diverse properties of NPs. The permeability is also effected by the properties of the vehicle and the structure and properties of skin along with their interactions ( Alikhan *et al*., 2009). Since the physical and chemical properties affecting dermal absorption of NPs are still undefined, permeability testing on a case-by-case basis is recommended ( ECHA, 2020).

Empirical studies have indicated that dermal absorption through diffusion is minimal for compounds with molecular weights exceeding 500 g/mol ( Buist *et al*., 2017). Consequently, this phenomenon can be regarded as negligible for nanoparticles (NPs) with molar masses on the order of 10 ^6^ g/mol and particle diameters around 10 nm. It has been shown that NPs can penetrate the skin via intra- or intercellular routes and transappendageal routes via hair follicles and sweat ducts ( Larese Filon *et al*., 2015). Particles that are not washed off generally remain in the upper layer of the epidermis, although there is evidence that some of the particles also reach the dermis. Potential risks associated with dermal exposure to insoluble NPs are ( Brouwer *et al*., 2016; Larese Filon *et al*., 2016; Larese Filon *et al*., 2015):

- ≤ 4 nm NPs can penetrate and permeate intact skin,
- 4 nm < *D _p_* ≤ 20 nm can potentially permeate intact and damaged skin,
- 20 nm < *D _p_* ≤ 45 nm can penetrate and permeate only damaged skin,
- > 45 nm NPs cannot penetrate nor permeate the skin.

Additionally, metallic NPs may dissolve and cause local effects or penetrate the skin in ionic form and cause systemic effects ( Zanoni *et al*., 2021). Impurities present in NPs can cause both localized and systemic adverse effects. Also, NPs permeability may be better for non-rigid NPs.

Estevan *et al.* (2022) estimated a risk related to wear of a face mask containing Ag NPs by the general population considering both daily use for 2 h/day and in workplace use for 8 h/day. The dermal exposure was calculated for the face mask with a contact surface area of 555 cm ^2^ face mask containing 113 μg-Ag/cm ^2^. It was assumed that Ag NPs in the textile are immediately released to skin surface. Dermal flux was set to correspond Ag NP penetration in a glycerolized human skin (3.8 ng/cm ^2^/h) that is similar to necrotic skin ( Bianco *et al*., 2014). Under these worst-case conditions described above, the risk ratio was ≤0.014 and the risk was adequately controlled. The approach is applicable for face mask safety assessment, but a more detailed approach is needed in a generic exposure scenario with longer wear times and larger contact surfaces by wearing other clothing containing Ag NPs.

In this study, a more realistic approach considering Ag release rates from textile to skin is considered rather than assuming instantaneous release and intake time trends. We demonstrate how Ag NP leaching from textile affects the Ag NP dermal exposure and uptake, while also assessing the role of unintentional oral exposure and the impact of hand/body washing in reducing exposure. The assessment was performed for antimicrobial textiles developed in Anticipating Safety Issues at the Design Stage of NAno Product Development (ASINA) for face mask wear and a full body exposure that is considered as the reasonable worst-case (RWC) exposure assessment. The simulations are performed for RWC for i) general population wearing face mask and ii) worst-case occupational exposure scenarios during wear of face mask and a full-body suit containing Ag NPs that are in contact with body with dermal exposure surface area of 20 000 cm ^2^ (2 m ^2^). Overall, by considering realistic exposure scenarios and incorporating factors such as leaching rates, unintentional exposure pathways, and the effectiveness of washing practices, the study aims to provide valuable insights into the potential risks associated with the use of Ag NP-containing textiles.

## 2. Methods

### 2.1 Dermal exposure model

Here non-volatile and inert NPs absorption to the skin via direct contact, and removal from the skin by wear and washing, and finger-to-mouth transfer mechanisms are considered. By considering these absorption, removal, and transfer mechanisms, the study aims to provide an understanding of fate and potential exposure pathways. The change in NP mass *m* (mg) on the skin surface over the exposure duration *t* (h) can be described with:


$$\frac{dm(t)}{dt}=S_{p}(t)–\left(k_{sc}+k_{o}+k_{ww}\right)\times m(t)\;(1)$$

Where *S _p_* ( *t*) (mg/min) is the NP application rate from a product *p* and rate constants *k _sc_* (1/min), *k _o_* (1/min) and *k _ww_* (1/min) respectively account for the NP losses from the skin surface by transfer to *stratum corneum* ( *sc*), inadvertent oral exposure ( *o*) and wear and washing ( *ww*). All rate constants are considered as net rates and the transport from receiving receptor back to the donor is insignificant.

***2.1.1 Emission source S _p_(t)***. The emissions from the product to skin can be described by using the product initial NP mass *m _p_* (mg) and NP transfer rate from the product to the skin as:


$$S_{p}(t)=k_{p}\cdot m_{p}\left(t\right)\cdot (1–e^{–k_{p}t}),\;(2)$$

Where the *k _p_* (1/min) is the first order loss rate constant and the product initial mass concentration *m _p_* ( *t*=0) = *m _p,0_* can be calculated from the amount of product in contact with skin *AP* (g), the product NP concentration *C* (mg/g) and the fraction of NPs available for dermal exposure *f _a_* (-), which is set to 1 corresponding to a situation where all NPs are available for dermal exposure.

Emission source for dermal exposure can be categorized according to the emission pattern as an instant-, continuous-, or varying emitter. For instant emission, all product is instantaneously in contact with skin (e.g., applying lotion), i.e., *k _p_* is very high and *S*( *0*) ≈ *m _0_*. For a continuous emitter, the transfer rate is small compared to the contact period and the mass loss from product is small, *i.e., m*( *t*) ≈ *m _0_*. Otherwise, the NP mass in the product decreases according to the emissions when assuming that there is no other loss mechanism than transfer to skin.

Indirect dermal exposure describes dermal exposure when skin is in contact with contaminated surfaces. This is described with an instant application source where the mass transfer is described with surface contamination *m _s_* (mg/cm ^2^), dermal contact area *A _d_* (cm ^2^) and transfer efficiency *TF* (-) estimating the fraction transferred from the contaminated surface to the skin.

***2.1.2 Assessment of the rate constants k _sc_, k _o_, and k _ww_.*** The rate constants can be estimated individually by measuring initial NP mass in the product and the NP mass in the receiving compartment. When *S*( *t*) = 0 and *x* is the only significant removal process, the rate constant for process *x* can be calculated as:


$$k_{x}=–\frac{1}{t}\;\text{ln}\;\left(1–\frac{m_{x}(t)}{m(t=0)}\right)\;(3)$$

Where *m* (mg) and *m _x_* (mg) are the donor and acceptor (receiving compartment) masses, respectively.

***2.1.3 Mass transfer via stratum corneum k _ss_ (intake)***. Ag NP penetration varies from 0.27×10 ^-6^ to 34×10 ^-6^ depending on the skin type ( Table 1). In this evaluation a precautionary assessment and penetration rate constants measured by Bianco *et al.* (2014) were used.

**Table 1.** Reported median penetration fluxes for Ag NPs under different conditions.

Study	Skin type	Exposure media	AgNP flux penetration, [ng/cm ^2^/h]	Lag time, [h]	Rate constant, *k _SC_*, [1/h]
( Bianco *et al.*, 2014)	Fresh human skin graft	Ag NPs (19±5 nm) in synthetic sweat using a Franz diffusion cell apparatus for 24 h. The donor solution surface load was 113 μg-Ag/cm ^2^.	0.2	8.2	1.8×10 ^-6^
	Cryopreserved human skin graft. Skin viability is reduced by 36% compared to fresh skin		0.3	10.9	2.7×10 ^-6^
	Glycerolized human skin graft. Comparable to necrotic skin from a morphological and structural point of view		3.8	6.3	34×10 ^-6^
( Larese Filon *et al.*, 2009)	Intact skin graft.	Ag NPs (25±7.1 nm) in synthetic sweat using a Franz diffusion cell apparatus for 24 h. The donor solution surface load was 70 μg-Ag/cm ^2^.	0.02	<1	0.27×10 ^-6^
	Damaged skin graft abraded according to the Bronaugh and Stewart (1985) protocol		0.10	<1	1.4×10 ^-6^

***2.1.4 Mass transfer via inadvertent oral exposure k _o_***. Two mechanisms are responsible for mouthing mediated ingestion:

1) Surface-to-hand contact followed by hand-to-mouth contact, and
2) direct mouthing of objects and ingestion of contaminants ( Li *et al*., 2021).

Relevant parameters for inadvertent oral exposure are surface loading (mg/cm ^2^), transfer efficiency (-), mouthing frequency (1/h) and contact surface area (cm ^2^). The transfer efficiency of NPs in hand-to-mouth contact is scarcely studied. Surface-to-hand studies showed that zinc oxide NPs transfer efficiency is *ca.* 30 times higher than for micron sized zinc oxide particles from metal and wood surfaces ( Brouwer *et al*., 2016). For powders, the hand-to-mouth transfer efficiency can be nearly complete ( Ng *et al*., 2012). The default surface area of both hands varies from 840 to 900 cm ^2^ for males ( OEHHA, 2008; te Biesebeek *et al*., 2014). Fingers mouthing frequency is studied in occupational environments ( Gorman Ng *et al*., 2016). Consumers finger mounting is studied only for children and infants: Surface area of fingers contacting accidentally mouth during fish tackle handling was estimated to be 19 cm ^2^ (three 3 fingertips and that each fingertip is 30% of the finger) and the contact frequency was assumed to be 9 times per hour ( OEHHA (2008). For adults, the average surface area for three fingertips per hand is 11.5 cm ^2^ (range: 9.3–14.4 cm ^2^; *n* = 12) ( Sahmel *et al*., 2015).

***2.1.5 Inadvertent oral ingestion, intake, and uptake***. Intake via inadvertent ingestion from hand to mouth contact can be estimated from the dermal load, contaminant transfer efficiency from hand to oral or perioral region and number of contacts ( Ng *et al*., 2012). In ingestion, the intake is assumed to be complete, *i.e. F _ret_* = 1, and the uptake can be estimated when the absorption of the substance from gastrointestinal tract is known. This model is applicable for solids, powders, pastes, gels and liquids including products with nanomaterials ( SCCS, 2019). The retention factor is usually poorly known, that is the main limiting factor considering the model reliability. NP absorption across intestine, *i.e.* uptake, can be calculated by using simplified mechanistic models when NPs permeability is derived from Caco-2 or Ussing chamber experiments ( Lundquist & Artursson, 2016; Ölander *et al*., 2016).

***2.1.6 Mass transfer via wear and washing k _ww_.*** NPs loss from skin by wear and washing is scarcely studied. Hand washing frequency in general population is *ca.* 8 times per day according to SDA (2005). NP removal efficacies by wear or washing are not available. Here, wear is not considered, and washing is simulated by using exposure time. These removal pathways can be applied when information on rate constants is available and if there is need for more detailed exposure assessment.

### 2.2 ASINA Ag NP textiles

Polystyrene textile (Klopman International, Vektron 8200; weight 145 g/cm ^2^) were coated with Ag NPs by using a spray coating system as described by Del Secco *et al.* (2022). The coating suspensions consisted of Ag (Sigma Aldrich, Milan, Italy) capped with hydroxyethylcellulose (Univar Solutions SpA, Milan, Italy) (AgHEC), hydroxyethylcellulose with changed molar ratio (AgHEC6.4) or adding curcumin (AgCur) as capping agent instead of HEC, all dispersed in water at concentrations of 0.1% w/w. The AgHEC, AgHEC6.4 and AgCur aqueous nano suspensions were produced by CNR- ISSMC (Faenza, Italy) using a patented production process (patent no. WO2016125070A1). Three different Ag NPs were applied to textiles by using the spray coating technique ( Table 2). The Ag concentration in each textile was measured by using inductively coupled plasma mass spectrometry according to ISO 11885:2007 (elemental concentration in different media).

**Table 2.** Textile coating parameters and Ag load in textile. Ag concentration on coating solution is 0.1 wt% for all samples. Plasma neutralization during spray coating is shown with (P).

Sample code	Embedding material	NM concentration on solution [wt%]	Embedding concentration on solution [wt%]	Flow rate [mL/min]	R2R speed [m/min]	Ag concentration [ng Ag/cm ^2^]
AgCurA	Curcumin	0.1	0.06	60	6	849 ± 31
AgCurB	Curcumin	0.1	0.06	80	6	1444 ± 203
AgCurC	Curcumin	0.1	0.06	60	4	1530 ± 151
AgCurC(P)	Curcumin	0.1	0.06	60	4	1147 ± 165
AgCurD	Curcumin	0.1	0.06	80	4	1957 ± 22
AgCurE	Curcumin	0.1	0.06	60	2	2258 ± 14
AgHEC6.4A	HEC6.4	0.1	1.07	60	6	1149 ± 144
AgHEC6.4B	HEC6.4	0.1	1.07	80	6	1222 ± 49
AgHEC6.4C	HEC6.4	0.1	1.07	60	4	1538 ± 41
AgHEC6.4C(P)	HEC6.4	0.1	1.07	60	4	1529 ± 82
AgHEC6.4D	HEC6.4	0.1	1.07	80	4	1861 ± 96
AgHEC6.4E	HEC6.4	0.1	1.07	60	2	2651 ± 399
AgHECC	HEC	0.1	0.92	60	4	1311 ± 671
AgHECC(P)	HEC	0.1	0.92	60	4	1406 ± 318
AgHECD	HEC	0.1	0.92	80	4	1574 ± 302

### 2.3 Textile washing

The washing simulation was based on the “ISO 105-C06 (A1S) for colour fastness to domestic and commercial laundering ( 2010)”. The tests were performed with a lab washing machine (Linitest Plus, Atlas Electrical Device); motor speed 40 ± 1 rpm, steel vessels (75 ± 5 mm diameter, 125 ± 10 mm height, 550 ± 50 mL) and a washing solution of 4 ± 0.01 g L ^-1^ ECE Colour Fastness Test Detergent 77 in deionized water (pH 10) and ten steel ball (Ø = 0.6 cm) to simulate the mechanical energy applied by friction to the textiles. Five pieces of each textile type were cut with an area of 40 cm ^2^ per piece and stacked in the vessel. A volume of 150 mL of washing solution was added to the vessel, together with the steel balls and the textiles. In order to do a conditioning of the washing solution inside the vessels at the same temperature as the washing machine, preheat of the washing solution at 40° C was done under stirring at 300 rpm for 30 min.

After the washing process, the washing water was collected, and the textiles were rinsed one by one dipping them five times in 10 mL of deionized water. Once this step was finished, the water was renewed to avoid accumulation of silver released in water. Finally, the textiles were placed on a paper, covered, and dried overnight at room temperature.

The washing water was filtered at different pore size (20 μm pore size, 0.45 μm pore size and 10 kDa, sequentially) and stored for electron microscopy characterization and silver quantification by using Inductively Coupled Plasma-Mass Spectrometry (ICP-MS). For the ICP-MS, 0.5 g of liquid sample is taken to perform an acid digestion with 5 ml of concentrated ultrapure nitric acid (HNO _3_ 70%) in an analytical microwave system at 250°C. Then, the obtained digestion residue is properly diluted to analyze the elements of interest by ICPMS. The quantification is performed by interpolation in a calibration curve prepared with commercial standards of the elements of interest. Electron microscopy images were treated by ImageJ ( Rasband, 2018)."

### 2.4 Abrasion tests

The simulation of a textile touching the skin was based on an adaptation of BS EN ISO 105-X12:2016 - Tests for color fastness Colour fastness to rubbing. The test was performed using a crock-meter (302-P, JBA). The equipment applies low energy wear on a 10 cm surface by a cylindric tip (ø = 16 mm) covered with cotton tissue. Before starting the experiment, calibration of the tip pressure was done by altering the weight until no pressure is applied on surface. Then, a 9 N weight was applied to the tip. Pieces of textile were cut at an approximate area of 40 cm ^2^ and stuck by double face tape into the dedicated spot for rubbing. A cotton napkin was dipped in a simulated sweat solution at pH 6.5 (0.5 wt% sodium chloride, 0.1 wt% lactic acid, 0.1 wt% urea) and rinsed for 1 min before covering the crock-meter tip. A total of 10 rubbing cycles (1 second/cycle) were performed by triplicates to unwashed textiles. The cotton napkin was replaced every 10 cycles. Characterization was performed to the cotton tissue (NM release receiving compartment) using ICP-MS.

Characterization was performed to the rubbered textiles (remaining NM concentration) and to the cotton tissue (NM release receiving compartment) using ICP-MS and SEM techniques. For the ICP-MS analysis (limit of detection of 5 mg NM/kg textile), all sample were weighed to perform an acid digestion with 3 mL of concentrated ultrapure nitric acid (HNO _3_ 70%) plus 1 mL of concentrated ultrapure hydrofluoric acid (HF 48%) in an analytical microwave system at 250°C. Then, the obtained digestion residue is properly diluted in order to analyse the element of interest (Ag) by ICPMS. The quantification is performed by interpolation in a calibration curve prepared with commercial standards of the elements of interest.

### 2.5 Risk characterization

Ag penetrated to *stratum corneum* is assumed to enter blood circulation immediately. Intake is normalized using 60 kg body weight (bw) according to the U.S. EPA (2011) recommendation. Estimated systemic derived-no-effect-limits (DNELs) ranged between 0.01 and 0.0375 mg/kg-bw/day for general population and between 0.02 and 0.075 mg/kg-bw/day for occupational settings ( Estevan *et al*., 2022). The risk characterization ratios (RCRs) were calculated by using bw-normalized daily intake divided with the lowest DNEL for general population (0.01 mg/kg-bw/day).

## 3. Results and discussion

### 3.1 Model comparison

Dermal model was compared with the dermal exposure model by von Goetz *et al.* (2013) They measured Ag releases for commercial Ag NP coated textiles in 30 min artificial sweat immersion and mechanical stress test based on modified ISO method 105-C06 for “color fastness to domestic and commercial laundering”. The release fractions and release constants calculated here (Table S1, *External data*; Koivisto *et al.*, 2024) were:

a) T-shirt, 83% polyester and 17% wool textile containing Ag 183 mg/kg: Release was 6.8% for acidic sweat and 5.0% for alkaline sweat corresponding to average release constant of 0.0017 1/min.
b) Trousers, 93% polyamide and 7% elastane containing Ag 41 mg/kg: Release was 13.2% for acidic sweat and 14.0% for alkaline sweat corresponding to average release constant of 0.0050 1/min.

Von Goetz *et al.* (2013) model is based on Ag leaching to artificial sweat and sweat generation that human generates during activity. The model calculates dermal exposure as Ag mass normalized with body weight by considering male and female physiological parameters (Table S1, *External data*; Koivisto *et al.*, 2024). Von Goetz *et al.* (2013) calculated dermal exposure for 60-min sport scenarios for male and female during wear of T-shirt and trousers having dermal contact area of 0.69 and 0.345 m ^2^, respectively. Average dermal exposure was 1235 and 659 µg for male and female, respectively, without body weight normalization.

**Figure 1.**
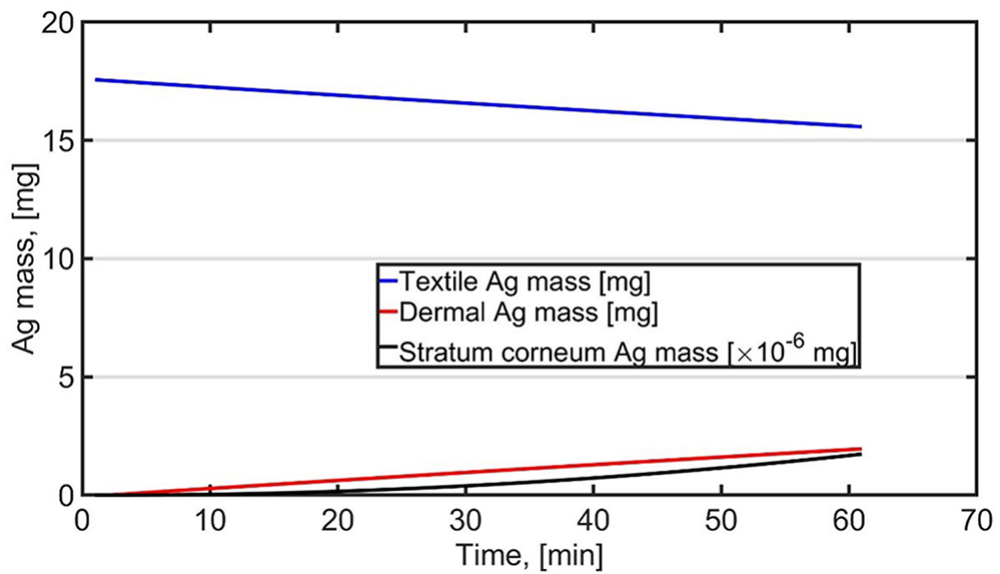
Dermal exposure and intake for female when wearing T-shirt and trousers during 60-min sport activity as presented by von Goetz *et al.* (2013).

We reproduced the sport scenario for female by using average release constants and assuming that the textiles and skin are sweaty during the 60-min period ( Figure 1 and Table S1, *External data*; Koivisto *et al.*, 2024). Dermal exposure was 2848 µg after 60-min exposure which is 4.3 times higher than average exposure calculated for von Goetz *et al*. (2013). Dermal intake was calculated by assuming that Ag absorption is the same as Bianco *et al.* (2014) reported for fresh skin. The intake was 2.6 ng corresponding to 0.043 ng/kg-bw (60-kg bw) corresponding to RCR of 4.3×10 ^-6^ (DNEL of 0.01 mg/bw-kg/day). Differences in dermal exposure are related to different approaches. Von Goetz *et al.* (2013) associate release to sweat generation while here the skin and textiles are considered as sweaty all times and release is independent of amount of sweat generated.

### 3.2 Soft abrasion release test

Release rate constant is calculated using Equation (3), where time is 10 sec, *m* corresponds to the initial Ag concentration and *m _x_* to the release for unwashed textiles. Concentration is equivalent to mass, as surface area is not changing through the release or washing. Release constants for unwashed textiles were 0.086, 0.12, and 0.15 1/min for AgCur, AgHEC6.4, and AgHEC textiles, respectively ( Table 3). The release constants obtained here are 25 to 44 times higher than average release rate constant measured by von Goetz *et al.* (2013) for acidic and alkaline sweat (0.0034 1/min). This can be explained by different textiles, coating techniques, and release test. It is also expected that the manufactured textile batch curation time was too short due to the manufacturing conditions that were designed primarily for evaluating process emissions and workers exposure under different operational conditions (unpublished).

**Table 3.** Ag release rate by abrasion for unwashed textiles (textile mass 145 g/m ^2^, release is for 10 second abrasion).

Sample code	Ag load in textile, ng/cm ^2^	Release to receptor, ng/cm ^2^	Release during 10 s, %	*k _a_*, [1/min]
AgCurA	849	17.6	2.1	0.13
AgCurB	1444	12.6	0.9	0.053
AgCurC	1530	19.1	1.2	0.075
AgCurC(P)	1147	17.9	1.6	0.094
AgCurD	1957	27.6	1.4	0.085
AgCurE	2258	31.7	1.4	0.085
**Average (standard deviation)**	**1531 (471)**	**21.1 (6.5)**	**1.4 (0.4)**	**0.086 (0.022)**
AgHEC6.4A	1149	25.0	2.2	0.13
AgHEC6.4B	1222	33.1	2.7	0.17
AgHEC6.4C	1538	34.1	2.2	0.13
AgHEC6.4C(P)	1529	16.4	1.1	0.065
AgHEC6.4D	1861	40.7	2.2	0.13
AgHEC6.4E	2651	47.3	1.8	0.11
**Average (standard deviation)**	**1658 (501)**	**32.8 (10.0)**	**2.0 (0.5)**	**0.12 (0.031)**
AgHECC	1311	35.9	2.7	0.17
AgHECC(P)	1406	23.3	1.7	0.10
AgHECD	1574	49.7	3.2	0.19
**Average (standard deviation)**	**1430 (442)**	**36.3 (8.9)**	**2.5 (0.5)**	**0.15 (0.033)**

### 3.3 Dermal exposure and intake via stratum corneum

Average release rate constants for unwashed textiles were used to calculate the intake and risk during wear of nano-Ag coated textiles ( Table 1). Calculation parameters for all scenarios are presented in Table S1 as *Extended data* ( Koivisto *et al*., 2024).

**Figure 2.**
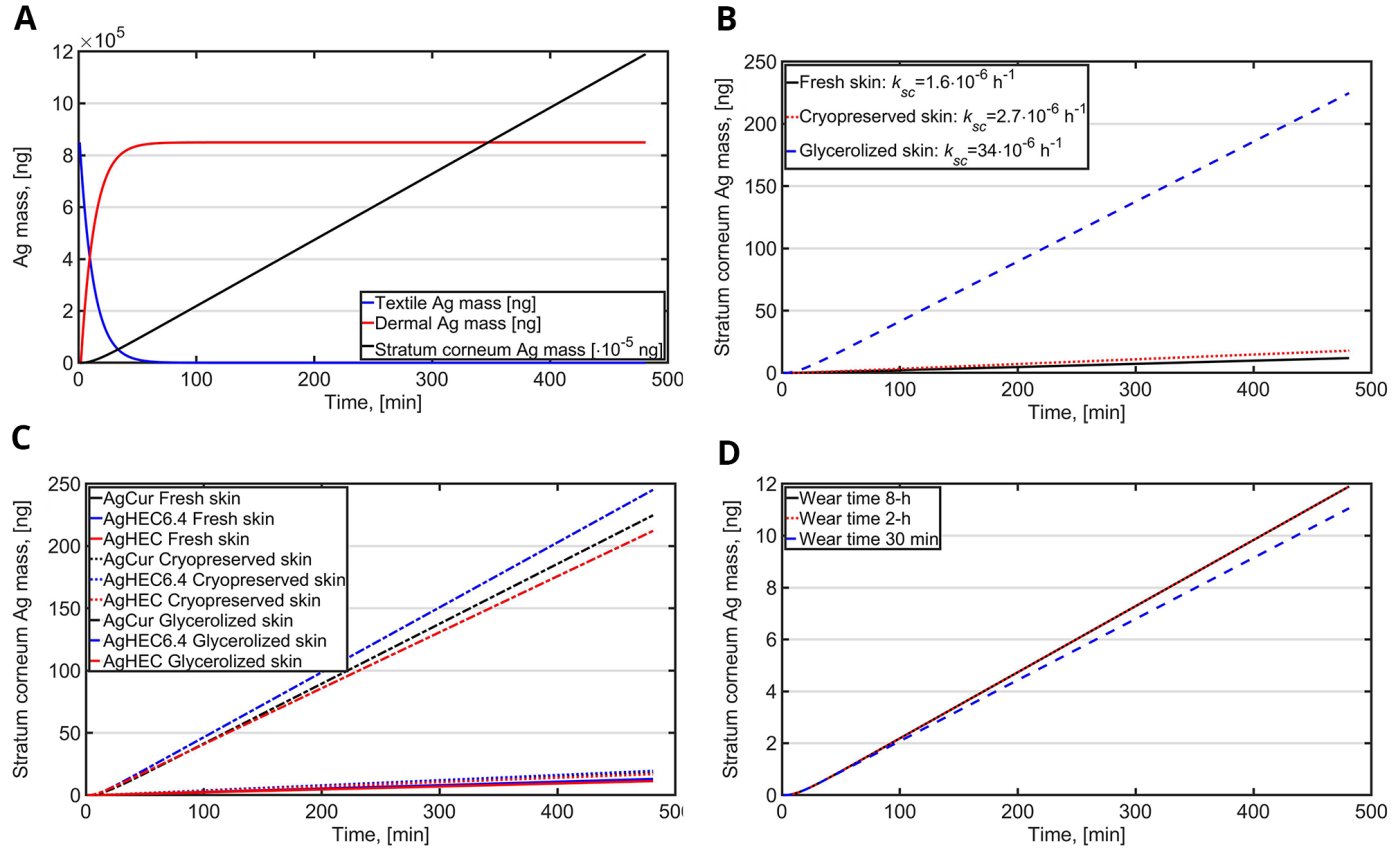
Dermal exposure and intake for face mask with surface area of 555 cm ^2^. **A**) An example of Ag release form AgCur coated textile to dermis and penetration to *stratum corneum* through fresh skin (healthy skin) during 8-h wear, **B**) An example of the effect of skin type on dermal penetration of Ag to *stratum corneum* for AgCur coated textile during 8-h wear, **C**) Dermal intake via *stratum corneum* during 8-h exposure for different textiles and for different skin types, and **D**) The effect of wear time (dermal exposure) for AgCur coated textile within 8-h duration for 8-h continuous wear, 2-h wear, and 30 min wear (fresh skin).

Release and intake of Ag NPs was calculated for 8-h wear of face mask for fresh skin, cryopreserved skin and glyzerolized skin ( Figure 2). During the first hour, 99% of the Ag NPs are released from the textile to skin. During 8-h wear in fresh skin 0.0014% is penetrated to *stratum corneum* ( Figure 2A). Absorption of Ag NPs in cryopreserved skin was 1.5 times higher than in fresh skin while the absorption for glycerolized skin was 19 times higher than in fresh skin ( Figure 2B). In fresh skin and cryopreserved skin, the absorption was in similar range ranging from 11 to 19 µg while for glycerolized skin the absorption was significantly higher ranging from 212 µg (AgCur) to 245 µg (AgHEC6.4 and AgHEC) ( Figure 2C). This was mainly caused by higher release rate constants because the average Ag NP initial mass concentrations were similar (1530, 1658 and 1430 ng/cm ^2^ for AgCur, AgHEC6.4 and AgHEC, respectively). The effect of wear time without hand washing was investigated for fresh skin during 8-h, 2-h and 30-min wear times ( Figure 2D). The 8-h and 2-h wear times resulted to similar intake levels and 30-min wear time reduced the intake by 1 ng.

RCR was investigated for 8-h wear of face mask (A=555 cm ^2^) and full body exposure (A=20 000 cm ^2^) ( Table 4). The highest RCR for face mask was 0.02 and for full body exposure 0.9 for glycerolized skin indicating adequately controlled exposure. Full body exposure for glycerolized skin representing necrotic skin is a worst-case exposure scenario and is an upper limit for exposure (tightly fitted clothing, sweating, and through-body abrasion with 10 N force for 8-h). Thus, it can be concluded that the Ag NP coated textile wear does not cause a risk related to Ag NP penetration through *stratum corneum*.

**Table 4.** Ag intake via *stratum corneum* during 8-h exposure and RCRs for different textiles and for different skin types. RCRs are calculated by using a DNEL of 0.01 mg/bw-kg/day (bw = 60 kg) for the general population.

Textile	Skin type			RCR (Intake/DNEL)		
	Fresh skin, [µg]	Cryopreserved skin, [µg]	Glycerolized skin, [µg]	Fresh skin	Cryopreserved skin	Glycerolized skin
Face mask, A = 555 cm ^2^						
AgCur	12	18	225	0.001	0.002	0.02
AgHEC6.4	13	19	245	0.001	0.002	0.02
AgHEC	11	17	212	0.001	0.002	0.02
Full body, A = 20 000 cm ^2^						
AgCur	429	643	8099	0.04	0.06	0.8
AgHEC6.4	468	702	8835	0.05	0.07	0.9
AgHEC	405	607	7647	0.04	0.06	0.8

### 3.3 Inadvertent oral intake

Inadvertent oral intake was investigated by assuming 1-h wear of AgCur treated gloves followed by finger mouthing three fingertips with surface are of 11.5 cm ^2^. During 1-h glove wear all Ag mass is released from the textile and the Ag mass in the three fingertips would be 1.8 µg. During the three fingertips mouthing and assuming complete NP transfer ( *k _o_* = 1 1/min) and gut absorption the RCR would be 0.0002. Compared to Ag dermal absorption via hands (900 cm ^2^; fresh skin), the 1-h glove wearing via *stratum corneum* would be 0.4 µg (RCR = 4×10 ^-5^) the finger mouthing cause 5 higher risk than dermal penetration. This demonstrates the importance of good hygiene practices.

### 3.4 Recommendations for development of dermal exposure assessment

NP release from textiles depends for example on the NP properties, application technique, e.g., incorporation or impregnation, textile type, concentration, and leaching media and mechanical stress ( Koivisto *et al*., 2017). In addition to synthetic sweat, other relevant exposure medias are saliva for oral exposure assessment and washing detergents for environmental and dermal exposure assessment. The absorption of Ag NPs differed significantly between skin conditions. Cryopreserved skin exhibited 1.5 times higher absorption compared to fresh skin, while glycerolized skin showed a much higher absorption rate, 19 times higher than fresh skin, attributed to higher release rate constants, despite similar initial mass concentrations of Ag NPs in the textiles. Overall, these findings highlight the complex interplay between release rates, skin condition, wear duration, and intake levels of Ag NPs from Ag coated textiles during wear. Distinction between dermal exposure and dermal intake should be made clear; von Goetz *et al.* (2013) reported dermal exposure normalized with body weight while here is reported dermal intake normalized by body weight that can be compared with the DNEL value when assuming that intake results to complete absorption to systemic circulation.

NP penetration through healthy skin is not a relevant exposure mechanism as compared to unintentional (peri-)oral exposure. Relevant and tailored experimental set-ups for measuring NP release from textiles and products are needed to understand which fraction can cause exposure and intake/uptake via oral route or through damaged skin. For face masks, it is expected that Ag intake via direct oral route is the main exposure pathway. Release to synthetic sweat is recommended to be used as surrogate source in dermal exposure assessment ( Koivisto *et al*., 2017). However, it is not clear how well the standards designed for textile wear resistance are applicable for exposure assessment. For example, it is expected that ISO 105 X12:2016 - Part X12: Colour fastness to rubbing does not represent typical use conditions when textiles/personal protective equipment are worn by workers or consumers. More realistic release test methods should be developed for mimicking typical/occupational use scenario. A systematic review of worst-case and RWC use conditions and hygiene practices would provide a better understanding of potential oral intake. Currently, the main development need in dermal exposure models are the contact surface area and retention efficiency of the pollutants ( Bogen *et al*., 2020).

Environmental exposure was not considered here. Washing experiments leachate Ag NP concentrations can be used to estimate environmental exposure over the textiles lifecycle ( Wimmer *et al.*, 2019). 

## Conclusions

This study investigates Ag NP release potential during washing and wearing. A mass balance model was used to describe the Ag NP dynamics between textile, dermis, stratum corneum, and (peri-)oral exposure. This model was applied for consumer exposure scenario but is also applicable for occupational exposure assessment via dermal contact of contaminated surfaces. The effect of Ag NP type, skin type and exposure duration to intake and risk was demonstrated. The modeling methods introduced here can be used to calculate realistic exposure estimates, which are relevant for example when estimating general population exposure for epidemiological studies. Ot the other hand, the model can be used to estimate realistic worst-case conditions to provide conditions of use for textiles containing NPs. Currently, the main limitation is to have a release test setup for NPs in textiles that mimic more realistic use conditions. The major risk associated with wear of Ag NP containing textiles was associated to gloves wear followed by unintentional finger mouthing.

## Ethics and consent

Ethical approval and consent were not required.

## Data Availability

Zenodo: Supplementary Material for "Exposure Assessment & Risks associated to wearing silver nanoparticle-coated textiles". https://zenodo.org/doi/10.5281/zenodo.10604889 ( Koivisto *et al*., 2024) This repository contains the following: Rate constants, exposure and intake calculation parameters, and model source code for Matlab R2018a (can be translated to Python). Supplementary material Data are available under the terms of the Creative Commons Attribution 4.0 International license (CC-BY 4.0).
