# Supplementary material for: Exposure assessment and risks associated with wearing silver nanoparticle-coated textiles
Source: Open Res Eur. 2024 Oct 2;4:100. Originally published 2024 May 15. [Version 2] doi: 10.12688/openreseurope.17254.2 (PMC11617820; doi:10.12688/openreseurope.17254.2)
Supplement: Peer review history

# Reviewer report: Reviewer response for version 2

**DOI:** 10.21956/openreseurope.20059.r46840

**Published:** 2024-12-18

**Authors:** Daniela Montalvo

**Affiliations:** 1 Trace Elements and Nanomaterials, Sciensano, Leuvensesteenweg, Tervuren, Belgium

This study provides a model that can be used to assess exposure to Ag NPs via dermal penetration or oral intake. The authors considered a realistic exposure scenario that includes tear and wear conditions. 

 - In general the methods are well describe and it is possible to replicate the study. However what I think is missing is information on the characterization of the textiles coated with Ag NPs. For example is it not indicated in the text  the size of the Ag NPS capped with cellulose and the particles coated with curcumin. Also an explanation why different capping agents were investigated? Were particles detected in the coated textiles? Electron microscopy imagines of the coated textiles can be included to support the data.

Is the study design appropriate and does the work have academic merit?

Yes

Is the work clearly and accurately presented and does it cite the current literature?

Yes

If applicable, is the statistical analysis and its interpretation appropriate?

Not applicable

Are all the source data underlying the results available to ensure full reproducibility?

Yes

Are the conclusions drawn adequately supported by the results?

Yes

Are sufficient details of methods and analysis provided to allow replication by others?

Partly

Reviewer Expertise:

Environmental chemistry, nanoparticles

I confirm that I have read this submission and believe that I have an appropriate level of expertise to confirm that it is of an acceptable scientific standard.

# Reviewer report: Reviewer response for version 2

**DOI:** 10.21956/openreseurope.20059.r46833

**Published:** 2024-12-04

**Authors:** Javier Esteban, Carmen Estevan

**Affiliations:** 1 Miguel Hernández of Elche University, Elche, Spain

The authors conducted a study aimed at characterising the risks associated with the use of Ag NP-containing textiles. The authors developed an exposure model for three scenarios i) face mask for general population, ii) face mask and a full-body suit for occupational exposure and iii) inadvertent oral intake (and combined scenario, see points 3 and 4). Subsequently, they carried out the pertinent risk characterisation. It is considered positive that multiple coatings were used to represent the variability of textiles available in the modelled scenarios. The article improves the knowledge of exposure assessment modelling and contributes to the risk characterization of nanoparticles in consumer products.

 Minor comments are provided below:

1. Conclusions (both abstract and main text). Please, consider to conclude on the basis of the quantitative RCR values. Please, report whether the risk was considered to be acceptable.
2. Page 4/17. Dermal exposure and NP size. Please, define Dp abbreviation. Insert Dp in “Dp < 4 nm …“ and in “Dp > 45 nm … “
3. Page 5/17. Paragraph starting with “In this study, a more realistic approach …”. State that you performed simulations for a hand-to-mouth scenario. E.g. *The simulations are performed for RWC for i) … … ii) … … and iii) inadvertent oral intake *… .
4. Page 5/17. Consider to simulate for combined exposure scenario consisted on dermal plus oral intake and report the outcome where corresponds.
5. Page 5/17. 2.1.3 Mass transfer via stratum corneum. Please, replace “kss” with “ksc”.
6. Page 6/17: Consumers finger “mounting” is studied only for children and infants. Please, replace “mounting” with “mouthing”.
7. Page 7/17. Table 2. Please, define R2R.
8. Page 7/17. Typo: replace “was doneunder stirring at 300 rpm for 30 min” with “was done under”.
9. Page 8 / 17. Regarding “Intake is normalized using 60 kg body weight (bw) according to the U.S. EPA (2011)”, please, include as well the EU guideline “Default human factor values for use in exposure assessments for biocidal products” from 2017. Web link: https://echa.europa.eu/documents/10162/1154636/recom_14+_default+human_factor_values_biocidal+products_en.pdf/88354d31-8a3a-475a-9c7d-d8ef8088d004
10. Page 8/17. Typo: replace “bw-kg”with “kg-bw”, i.e. *“…(DNEL of 0.01 mg/ *kg-bw */day)”*.
11. Fig 1. Please, consider to add a figure caption. For example, Dermal exposure and intake were calculated by using the dermal exposure model developed in this study. For details, see section 3.1 Model comparison.
12. Page 9/17. Regarding the sentence *“The highest RCR for face mask was 0.02 and for full body exposure 0.9 for glycerolized skin indicating adequately controlled exposure”*, it is debatable that a RCR of 0.9 indicate an adequately controlled exposure. Please, consider to split the sentence in two, one for face mask and another one for glycerolized skin.
13. Pages 9 and 10/17. Regarding necrotic skin, “Full body exposure for glycerolized skin representing necrotic skin is an unrealistic exposure occupational scenario”. Please, consider to elaborate on worst-case assumptions leading to RCR values of 0.8-0.9. Regarding the sentence “Thus, it can be concluded that the Ag NP coated textile wear does not cause a risk related to Ag NP penetration through stratum corneum”, consider to replace “not cause a risk” with “causes an acceptable risk”.
14. Page 10/17. Figure 2C. The type of the line (dotdash) is barely seen in the legend. Improve reporting of legend.

Is the study design appropriate and does the work have academic merit?

Yes

Is the work clearly and accurately presented and does it cite the current literature?

Yes

If applicable, is the statistical analysis and its interpretation appropriate?

Yes

Are all the source data underlying the results available to ensure full reproducibility?

Yes

Are the conclusions drawn adequately supported by the results?

Yes

Are sufficient details of methods and analysis provided to allow replication by others?

Yes

Reviewer Expertise:

Exposure assessment, nanoparticles, biocides assessment

We confirm that we have read this submission and believe that we have an appropriate level of expertise to confirm that it is of an acceptable scientific standard.

# Reviewer report: Reviewer response for version 2

**DOI:** 10.21956/openreseurope.20059.r44809

**Published:** 2024-10-10

**Authors:** Gülşah Ekin Kartal

**Affiliations:** 1 Dokuz Eylül University, İzmir, Turkey

The previously mentioned revisions have been made. The manuscript can be accepted in its current form.

Is the study design appropriate and does the work have academic merit?

Yes

Is the work clearly and accurately presented and does it cite the current literature?

Partly

If applicable, is the statistical analysis and its interpretation appropriate?

Partly

Are all the source data underlying the results available to ensure full reproducibility?

Partly

Are the conclusions drawn adequately supported by the results?

Yes

Are sufficient details of methods and analysis provided to allow replication by others?

Yes

Reviewer Expertise:

Textile chemistry, nanotechnology, microencapsulation, fishing nets, ecological textiles

I confirm that I have read this submission and believe that I have an appropriate level of expertise to confirm that it is of an acceptable scientific standard.

# Reviewer report: Reviewer response for version 1

**DOI:** 10.21956/openreseurope.18646.r41677

**Published:** 2024-07-09

**Authors:** Sandra Velasco-Ramírez, Ana Cristina Ramírez Anguiano

**Affiliations:** 1 Chemistry Department, School of Exact Sciences and Engineering, University of Guadalajara, Guadalajara, Jalisco, Mexico; 2 Chemistry Department, School of Exact Sciences and Engineering,, University of Guadalajara, Guadalajara, Jalisco, Mexico

**1. Introduction and Background:**

 The introduction provides a comprehensive overview of the context and importance of the study. The relevance of investigating textiles coated with silver nanoparticles is well established, given their increasing use and potential health implications.

Recommendation:

 Although the introduction is comprehensive, it could benefit from a more detailed discussion on the regulatory landscape and existing guidelines or standards for silver nanoparticles in textiles. This would provide a clearer picture of the current framework in which this study is taking place.

**2. Methodology:**

 The methodology section is well detailed, describing the experimental design, sample preparation and analytical techniques used. The use of multiple assessment methods improves the reliability of the findings.

Recommendation:

  Ensure that reproducibility of methods is well documented. Providing supplementary material with detailed protocols or referring to standard procedures would be beneficial for other investigators wishing to replicate the study.

**3. Exposure Assessment:**

 Exposure assessment is exhaustive, using both in vitro and in vivo models to assess potential risks. The choice of models and endpoints is appropriate for assessing dermal exposure to nanoparticles.

Recommendation:

 The study should be strengthened by including a wider range of exposure scenarios, such as different lengths and frequencies of textile use. In addition, considering the possibility of nanoparticle release during washing and subsequent exposure could provide a more comprehensive view of the risks.

**4. Risk Assessment:**

 The risk assessment framework is solid, incorporating hazard identification, dose-response assessment, exposure assessment and risk characterization. The use of quantitative approaches to estimate risk levels is laudable.

Recommendation:

 To improve risk assessment, probabilistic hazard assessment should be considered. This would take into account variability and uncertainty in exposure and toxicity data, providing a more qualitative understanding of potential risks.

**5. Results and Discussion:**

 The results are clearly presented, with appropriate use of tables and figures to illustrate key findings. The discussion effectively interprets the results in the context of the existing literature, highlighting the novel contributions of the study.

Recommendation:

 Make sure that the discussion section critically evaluates the limitations of the study, such as possible confounding factors or uncertainties in the data. In addition, suggesting specific directions for future research based on the study's findings would be valuable in advancing the research field.

**6. Conclusion:**

 The conclusion briefly summarizes the main findings and their implications.

 It effectively communicates the significance of the study and its contribution to the understanding of nanoparticle exposure and risk.

Recommendation:

 The conclusion could be expanded to include recommendations based on the findings. This addressed to stakeholders, such as manufacturers, regulators and consumers as well. Providing actionable insights may enhance the practical impact of the report.

**7. General Comments:**

- Innovation:

  The study approaches a key gap in the literature regarding the safety of textiles coated with silver nanoparticles, showing innovative approaches to exposure and risk assessment.

Clarity and Readability:

 The manuscript is well written and structured, making it accessible to a broad reading group, including researchers, policy makers and industry stakeholders.

**Aspects Not Evaluated:**

Supplementary Materials:

 If the study includes supplementary materials, they were not reviewed as part of this assessment. Ensuring that these materials are complete and accessible is important for full transparency and reproducibility.

Long Term Exposure:

 The assessment focuses on acute and sub-chronic exposure scenarios. Long-term exposure effects, including possible chronic health outcomes, were not evaluated and represent an important subject area for future research.

Environmental Impact:

 The study focuses mainly on risks to human health. Assessing the environmental impact of the release of silver nanoparticles from textiles during use and disposal is another crucial aspect that deserves further investigation.

  As a whole, the study is a highly valuable contribution to the area of research, providing valuable insights into the risks associated with textiles coated with silver nanoparticles. Addressing the recommendations provided may further improve the robustness and impact of the research.

Is the study design appropriate and does the work have academic merit?

Yes

Is the work clearly and accurately presented and does it cite the current literature?

Yes

If applicable, is the statistical analysis and its interpretation appropriate?

Yes

Are all the source data underlying the results available to ensure full reproducibility?

Partly

Are the conclusions drawn adequately supported by the results?

Yes

Are sufficient details of methods and analysis provided to allow replication by others?

Yes

Reviewer Expertise:

ImmunochemistryInflammationImmunology of Infectious DiseasesNatural Product ChemistryNatural Product PharmacologyExtraction of Natural ProductsMedical NanotechnologyAnalytical ToxicologyAcute Toxicity TestsEcotoxicologyEnvironmental NanotechnologyFood NanotechnologyAntioxidantsSecondary Metabolites

We confirm that we have read this submission and believe that we have an appropriate level of expertise to confirm that it is of an acceptable scientific standard, however we have significant reservations, as outlined above.

## References

[1] : Health Impact of Silver Nanoparticles: A Review of the Biodistribution and Toxicity Following Various Routes of Exposure. *Int J Mol Sci* .2020;21(7) : 10.3390/ijms21072375 10.3390/ijms21072375 32235542 PMC7177798

[2] : The Potential of Silver Nanoparticles for Antiviral and Antibacterial Applications: A Mechanism of Action. *Nanomaterials (Basel)* .2020;10(8) : 10.3390/nano10081566 10.3390/nano10081566 32784939 PMC7466543

# Reviewer report: Reviewer response for version 1

**DOI:** 10.21956/openreseurope.18646.r41685

**Published:** 2024-07-03

**Authors:** Gülşah Ekin Kartal

**Affiliations:** 1 Dokuz Eylül University, İzmir, Turkey

As a reviewer for this article on exposure assessment and risks associated with wearing silver nanoparticle-coated textiles, here are some suggestions for corrections and comments on the article in general: Provide more details on the methodology used for exposure assessment, including the sampling techniques, analytical methods, and any limitations encountered during the study. Clarify how the exposure levels were measured and assessed in relation to silver nanoparticle-coated textiles. Discuss the implications of the findings in relation to existing literature on the topic and highlight any novel contributions of the study. Please add more recent articles about silver nanoparticles in textile. Provide a comprehensive discussion on the potential health risks associated with wearing silver nanoparticle-coated textiles, considering both dermal and inhalation exposure pathways. Summarize the key conclusions of the study and highlight the significance of the research in the field of nanotechnology and textile safety. The article could benefit from more detailed information on the role of silver nanoparticles in textiles, their potential release mechanisms, and their interaction with the human body. Consider expanding the discussion on the regulatory aspects related to the use of nanomaterials in consumer products and the need for standardized safety assessments. Overall, the article provides valuable insights into the exposure assessment and risks associated with silver nanoparticle-coated textiles. By addressing the suggested corrections and enhancing the clarity and depth of the content, the article can further contribute to the understanding of nanotechnology applications in the textile industry and their implications for human health.

Is the study design appropriate and does the work have academic merit?

Yes

Is the work clearly and accurately presented and does it cite the current literature?

Partly

If applicable, is the statistical analysis and its interpretation appropriate?

Partly

Are all the source data underlying the results available to ensure full reproducibility?

Partly

Are the conclusions drawn adequately supported by the results?

Yes

Are sufficient details of methods and analysis provided to allow replication by others?

Yes

Reviewer Expertise:

Textile chemistry, nanotechnology, microencapsulation, fishing nets, ecological textiles

I confirm that I have read this submission and believe that I have an appropriate level of expertise to confirm that it is of an acceptable scientific standard, however I have significant reservations, as outlined above.
